# The side effects of service changes: exploring the longitudinal impact of participation in a randomised controlled trial (DOORWAYS) on staff perceptions of barriers to change

**DOI:** 10.1186/s12888-019-2370-6

**Published:** 2019-12-18

**Authors:** Caroline Laker, Matteo Cella, Deborah Agbediro, Felicity Callard, Til Wykes

**Affiliations:** 1 Faculty of Health, Education, Medicine and Social Care, Anglia Ruskin University, Chelmsford Campus, Bishop Hall Lane, Chelmsford, Essex CM1 1SQ UK; 2 Department of Psychology, King’s College London, Institute of Psychiatry, Psychology & Neuroscience, PO77, Room 2.11, London Henry Wellcome Building, 16 De Crespigny Park, London, SE5 8AF UK; 3 Institute of Psychiatry, Psychology & Neuroscience, King’s College London, London, UK; 4 Birkbeck, Department of Psychosocial Studies, University of London, Malet Street, Bloomsbury, London, WC1E 7HX UK; 5 South London & Maudsley NHS Foundation Trust, Bethlem Royal Hospital, Monks Orchard Road, Beckenham, BR3 3BX UK

**Keywords:** Mental health wards, Nurses’ perceptions, Barriers to change, Unstructured multivariate linear regression models

## Abstract

**Background:**

Staff and service users have expressed concerns that service improvements in British mental health wards have been slow or transient. It is possible that certain changes are positive for some (e.g. service users), but negative for others (e.g. staff), which may affect implementation success. In this study, we explore whether a programme of change to improve the therapeutic milieu on mental health wards influenced staff perceptions of barriers to change, 12 months after implementation.

**Method:**

A cluster randomised controlled trial called DOORWAYS was conducted on eight British, inner-city acute mental health wards. Randomisation was achieved using a list randomly generated by a computer. A psychologist trained ward staff (mainly nurses) to deliver evidence-based groups and supported their initial implementation. The impact of these changes was measured over 12 months (when 4 wards were randomised), according to nurses’ perceptions of barriers to change (VOCALISE), using unstructured multivariate linear regression models. This innovative analysis method allows maximum use of data in randomised controlled trials with reduced sample sizes due to substantial drop out rates. The contextual influences of occupational status (staff) and of workplace setting (ward) were also considered.

**Results:**

Staff who participated in the intervention had significantly worse perceptions of barriers to change at follow up. The perceptions of staff in the control group did not change over time. In both groups (*N* = 120), direct care staff had more negative perceptions of barriers to change, and perceptions varied according to ward. Across time, direct care staff in the intervention group became more negative than those in the control group.

**Conclusion:**

Participation in this program of change, worsened staff perceptions of barriers to change. In addition, occupational status (being from the direct care group) had a negative effect on perceptions of barriers to change, an effect that continued across time and was worse in the intervention group. Those providing direct care should be offered extra support when changes are introduced and through the implementation process. More effort should be placed around reducing the perceived burden of innovation for staff in mental health wards.

**Trial registration:**

ISRCTN, ISRCTN 06545047. Registered 29/04/2010, https://www.isrctn.com/search?q=06545047

## Background

Despite global investment into the development of new innovations, relatively few research findings are translated into health care practice [1–3]. British acute mental health wards, in particular, have attracted criticism over recent decades for being slow to deliver improvements in care [4, 5], and for low levels of activity in promoting social engagement and therapeutic interaction [6, 7]. More research is clearly needed to examine what prevents such changes from being delivered successfully in these settings.

To explore barriers to change in mental health wards, this study will examine the impact of a programme of change on staff perceptions 12 months after its implementation. It should be noted that in the U.K. the term “ward” is commonly used with no negative connotation to refer to an in-patient setting. In this study, “ward” was the term used in the setting where the study was conducted, and as such, will be used to describe the in-patient settings that participated.

### Why are improvements needed in mental health wards?

The changes which provided the basis for this study were delivered as part of DOORWAYS, a U.K. National Institute for Health Research funded clinical trial (which is described in more detail later). DOORWAYS was conducted in response to concerns expressed by both patients and staff that acute in-patient wards provide poor quality care, with limited access to activities with an established evidenced base, and insufficient therapeutic interaction [8, 9]. Indeed, some in-patients have expressed concerns over their safety on mental health wards, with staff who appear too busy to listen to their problems, poor communication and unnecessary reliance on coercive interventions, all factors which damage the nurse/patient relationship [5, 10]. Given these criticisms and wide concerns that improvements have been not gone far enough [7], more empirical evidence is needed to understand what supports and prevents changes to move this important area of mental health care forwards. DOORWAYS also investigated the sustainability of positive effects by examining the whether exposure to the programme and the number of increased activities affected staff morale.

### What might prevent successful change in acute mental health wards?

In their article [11], Powell et al. argued in favour of linking implementation strategies to barriers that consider the context of the setting involved. This is important because any impact from health innovations is likely to be influenced by social and organisational factors, which may either enhance or hinder implementation [12]. In the U.K. (and across Europe) mental health care is now largely delivered in the community after a 20 year process of de-institutionalisation [13]. As a result, service users are often admitted to mental health wards in acutely unwell states and nurses spend more time responding to crises and less time in therapeutic engagement, a situation which may impede implementation processes [8, 9, 14, 15]. Mental health wards are dynamic environments with frequent shift rotations of staff and a rapid patient turnover in response to increased demand for beds inner-city areas where demand for beds is high [16–18]. Even before introducing changes, these complex environments are prone to volatility and disruption because the client group are acutely unwell and often distressed [19], and may be affected either personally or vicariously by the Mental Health Act (2007), which can lead to detention, enforced medication and in some cases to violence [20].

As well as considering the environment, the views of nursing staff are key because nurses are the largest staff group working in the National Health Service (NHS). As such, nurses can make a significant contribution to the development and running of the services. In mental health wards, nursing staff are responsible for co-ordinating most of the ward activities. They deliver daily care through extensive interactions with those who are admitted as service users. Their cooperation is essential if ward level changes are required. However, research that explores why mental health staff might have difficulties when incorporating innovation into practice is lacking [21, 22], and the longitudinal impact of changes on staff working in mental health wards has not yet been explored.

Moreover, intensive programmes of change are likely to bring disruption before improvements, and as it is generally well accepted that employees thrive on stability and resist changes that cause uncertainty and disruption [23, 24], it may be that certain staff in mental health wards find changes harder to cope with than they might, in a more stable setting. This may depend on where they are positioned in the organizational hierarchy of the ward. In the general health literature there is evidence that nurses respond differently to changes according to their occupational status, with managers having more positive responses to change than more junior staff [25]. In our previous studies, we found that occupational status predicted perceptions of barriers to change in mental health ward staff [26, 27]. This study will develop that finding by examining whether staff perceptions in two different occupational categories (senior staff and direct care staff) changed over time, as a result of participation in DOORWAYS.

Although it is clear that changes are challenging in mental health wards, there is little evidence to support strategies for change in these settings. To minimise potentially damaging impacts on staff morale and on the nurse/patient relationship when changes are made, more empirical evidence is required which explores how changes affect staff. This will support the development of evidence-based strategies which empower staff to make changes that take the environment into account, as part of the process. There is some evidence that ward climate adversely affects how mental health nursing staff respond to changes [17]. Our previous work also showed that ward climate affects how staff perceive barriers to change in mental health wards [28]. However, there is a particular lack of evidence which takes the longitudinal effects on changes on staff into consideration. This study develops current evidence by exploring whether different ward settings influenced the perceptions of the staff who worked there, across time. Taking a longer term view of the effects of change on staff may help inform why changes have been difficult to embed in mental health wards.

## Methods

### Aims

This study contributes to the field of implementation science by investigating whether nursing participation in a programme of change affected their perceptions of barriers to change. The effects of occupational status and ward on perceptions of barriers to change will also be considered.

### Hypothesis

After controlling for ward and occupational status, perceptions of barriers to change will be more negative in nursing staff who participated in DOORWAYS compared to those in the control group.

#### Study context and trial design

This study uses longitudinal data from nursing staff working within seven inner city, acute in-patient wards, and one specialist in-patient women’s service, in a mental health National Health Service Foundation trust. These data were collected for the DOORWAYS trial [8, 9], which was funded and ran from February 2008 until April 2010.

DOORWAYS was stepped wedge, cluster randomized controlled trial (RCT) which was designed so that at each randomisation point, two wards were assigned to the therapeutic intervention arm and those on the ‘waiting list’ provided a control. Data were collected pre-randomisation, so that randomised wards also acted as controls. The order of ward allocation to the intervention was determined by a randomly generated list, which was computed by a statistician using the *ralloc* procedure in Stata, which is a statistical software package.

DOORWAYS tested the impact of a range of evidence based interventions to improve the therapeutic milieu in eight mental health wards, by training nurses and occupational therapists to deliver mainly cognitive behaviour therapy (CBT) based groups [8, 9]. The groups were selected because there is evidence provided by the National Institute for Clinical Excellence (U.K.) of their efficacy in improving outcomes for people with depression/anxiety/psychotic/personality disorders, these being the most commonly found conditions on the wards involved [5, 9]. There was also a period of consultation with the clinical leads, ward managers and nursing staff on each ward to establish which interventions would be most suitable, based on the specific needs of their clients [8, 9].

#### Intervention

Staff who participated in delivering the groups were qualified mental health nurses, which requires a BSc in mental health nursing. This initial training exposes nurses to basic information about therapeutic interventions and assess students’ ability to deliver brief interventions, often using an objective structured clinical examination (OSCE) approach. This adequately prepared them for the additional training after randomisation, delivered by the DOORWAYS clinical psychologist in a number of groups and evidence base activities which included:

- Communication skills – to improve communication between staff and service users, and communication more generally.
- Social Cognition & Interaction Training – to improve service users’ understanding of social situations and minimise misunderstandings with others [29].

Wards were also offered a choice of therapeutic activities, which they selected based on their service requirements, as follows:

- Hearing Voices Group - to reduce the distress associated with hearing voices, and to teach new coping skills whilst improving self-esteem [30].
- Self Esteem and Coping with Stigma - to reduce the stigma associated with mental health problems, including the negative self-evaluations which may maintain low self-esteem [31].
- Emotional Coping Skills - to teach skills to service users for coping with overwhelming negative emotions (common in those who self-harm) [32]. This group was based on dialectical behavioural therapy.
- Relaxation Techniques – to teach progressive muscle relaxation techniques and breathing exercises to service users in preparation for sleep [31]
- Problem Solving Skills – to teach structured methods for problem-solving and involved identifying the problem, brain storming possible solutions, and selecting the best solution(s) [33].

Implementation followed a change management strategy adapted from ‘Diffusion of lnnovations’ [34]. The aim was to identify enthusiastic individuals as champions, which would motivate other members of the team to adopt the intervention. After six months the groups were expected to run regularly because a majority of staff had been trained and involved. After the training, there followed a process of establishing the groups on the wards, through demonstrations by the psychologist. The nursing staff were then asked to deliver the groups independently by the third month. Until the end of six months the psychologist was available for advice and ongoing support. By the twelfth month, all four wards included in this study had received training in communication skills and the intervention groups were running as outlined in Table 1.

**Table 1:** Training status at twelve months

Ward 3	Ward 4	Ward 5	Ward 8
Cognitive Remediation Therapy	Emotional Coping Skills	Cognitive Remediation Therapy	Cognitive Remediation Therapy
Social Cognition & Interaction Training	Problem Solving Skills	Problem Solving Skills	Problem Solving Skills
Hearing Voices		Hearing Voices	Hearing Voices
Relaxation Techniques			Emotional Coping Skills
Self Esteem & Coping with Stigma			

The DOORWAYS trial provided a vehicle to test whether perceptions of barriers to change worsened over time as a result of implementation disruption. On the four wards that had implemented DOORWAYS interventions, 12 months was considered sufficient exposure time to have affected staff perceptions. At 12 months, two wards had been delivering the interventions for 12 months and 2 wards had been delivering the interventions for 6 months. To be clear that staff perceptions had changed because of change related disruption, a control group, who were not exposed to any programme of change were required. At 12 months, 4 wards had not yet started the DOORWAYS intervention, providing an equal number of control wards for comparison to those that had received the intervention.

#### Inclusion criteria

All permanently employed nursing staff on acute in-patient wards were eligible to take part in this stage of the study, including staff from band seven (team leaders), band six (clinical charge nurses), band five (entry level qualified staff) and band three (health care assistants).

#### Sample size

To estimate the number of participants necessary for multi-level regression models we followed the general rule suggested by Green [35] of ten cases per variable. Given *N* = 120 participants were included in a regression model with five variables, this sample was sufficient.

#### Ethics, consent and permissions

A local NHS Research Ethics Committee (07/H0809/49) awarded ethical approval for this study. Participants were provided with information sheets and given time to consider participation before providing written, informed consent.

#### Procedure

Staff were recruited to each time point over 30 days by an on-site team of research assistants. All staff measures were completed by self-report. Although it was possible for the same staff to participate at multiple time points, changing shift patterns meant that those who participated at baseline were not necessarily available at follow up, leaving the dataset susceptible to losses. The baseline data were collected in March and April 2009 and the follow up data were collected 12 months later.

#### Measures

##### Primary outcome measure: staff perceptions of barriers to Change

As outlined in our previous papers [26, 27], the 18 item Views Of Change and Limitations in In-patient Settings (VOCALISE) measure [26] was developed with contributions from mental health nurses to capture their views of working in wards, and multiple causes of resistance to change. Some “barriers to change” identified below as original items from the VOCALISE measure [27]) reflected organisational difficulties:

- When it comes to change, information is not circulated effectively on my ward;
- I’m too busy to keep up to date with information about the changes that are happening on my ward;
- Poor leadership prevents changes happening on my ward
- Inadequate staffing prevents changes being successful on my ward.

Some described staff reluctance and withdrawal:

- When some staff stop engaging with planned changes resistance spreads through my whole team
- I feel disheartened when others do not want to get involved in changes.

VOCALISE is scored using a Likert scale of six options which included strongly agree, slightly agree, agree, disagree, slightly disagree and strongly disagree. The highest score is 108, and the lowest is 18. In this study, high VOCALISE scores indicated negative perceptions of barriers to change.

##### Secondary outcome measures

- *Occupational status:* as also outlined in a previous paper [27] this variables had two groups 1) direct care staff and 2) managers. Direct care staff were healthcare assistants and band 5 qualified nursing staff. Managers were bands six and seven nursing staff (i.e. clinical charge nurses, practice development nurses and team leaders).
- *Ward: an eight-category “ward” variable was used to determine whether staff perceptions of barriers to change were different according to the ward staff were working in, hence in this study ward (as a fixed effect) is understood as a proxy measure for ward climate.*
- *Time:* two time points were included (baseline, 12-month follow-up).
- *Treatment group:* two groups participated: (intervention and control).

#### Unstructured multivariate linear models

As there were a large number of missing data at follow up in this study (only 43% of the baseline sample were repeat participants), innovative unstructured multivariate linear models were adopted.

Unstructured multivariate linear models use both baseline and follow-up data as the correlated outcome, enforcing a zero treatment effect at baseline, with an unstructured covariance matrix for baseline and follow-up measures [36–40]. The models allow more information from the data to be used (compared to the traditional ANCOVA model), by also including the individuals who have no outcome measurement, but who do have a baseline measurement. Furthermore, unstructured multivariate linear models also deal with partially missing baseline measurements in RCT’s in the most statistically efficient way when the outcome is measured [39].

Unstructured multivariate linear models are advantageous because they can handle substantial drop out rates in RCT’s in an unbiased way under a ‘missing at random’ (MAR) assumption [41]. Under this assumption, whether participants withdraw from the trial or remain, the distribution of their data is conditionally the same, because their unobserved future is based on their observed past [42]. This approach is also preferable to using multiple imputation, a less efficient form of this type of analysis, since the two approaches broadly coincide, as the number of imputations gets large.

#### Analysis strategy

**Fig. 1:**
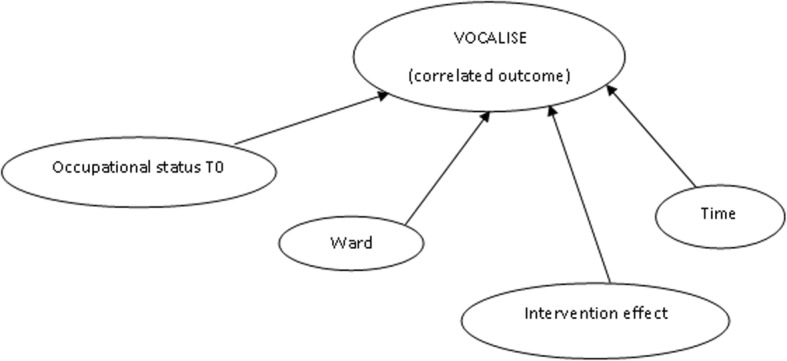
The potential negative intervention effect on staff perceptions of barriers to change when including covariates time, occupational status and ward

Any impact of the DOORWAYS intervention at follow up on staff perceptions of barriers to change will be investigated using an unstructured multivariate linear model. In the model, the correlated outcome variable will be staff perceptions of barriers to change (VOCALISE) and the main predictors of interest (included as fixed effects) will be *time* and *treatment group* (see Table 2). In our previous papers, we showed that ward climate and occupational status affected perceptions of barriers to change [27, 28]. In this study, ward and occupational status will therefore be included as covariates in the model (see Fig. 1):

**Table 2:** Considerations for the interpretation of the unstructured multivariate linear model

VOCALISE	Outcome
Intervention effect	Estimates the difference in group scores at follow up, adjusted for all other included covariates. If coded to provide estimates for those who participated in the intervention wards, it assumes an interaction between group and time because this variable comprises 2 groups: 1) those who were in the control group at T1 and 2) everybody else (baseline sample and those who did receive the intervention at T1). Hence: $$ intervention\ effect=\left\{\begin{array}{cc}\ 1& ifstudy\ group\ is\ control\ (0)\ and\ time\ is\ 1\\ {}\ 0& elsewhere\end{array}\right. $$ $$ interventioneffect=\Big\{{\displaystyle \begin{array}{cc}1& ifstudygroupisintervention(1) andtimeis1\\ {}0& elsewhere\end{array}}\operatorname{} $$
Time	The models do not measure a main effect of time because as discussed above, an interaction between time and the intervention effect variable is assumed. The time variable allows an estimate of the adjusted change in the outcome between baseline and follow up. By changing the coding in the intervention effect variable, the estimates for the time variable are also restricted to the control group only or the intervention group only. As there is an interaction between group and time in the intervention effect variable, the effects within each treatment group are expected to be different over time.
Ward & Occupational status	The estimates for ward and occupational status are the mean outcome score differences between the different categories of ward and occupational status across time, given the assumption that both arms of the trial started with the same scores at T0. Therefore, for example, the estimate for occupational status is the mean score difference between the two categories of occupational status, adjusting for ward, time, and the intervention effect that forces the mean scores to be the same at baseline. The estimates for ward and occupational status are across time, and are not changed by recoding the variable for intervention effect.
Constant	The constant represents the estimated mean outcome score. As the models adjust for occupational status, this score is based on occupational status = 0 (direct care); and the reference category for ward, which was ward 1, where staff had the most negative perceptions of barriers to change. The constant is the same whether the intervention effect variable is coded to represent those who did, or those who did not receive the intervention because of the coding (which enforces a 0 treatment effect at baseline in order to meet the assumptions of an RCT).

The interpretation of unstructured multivariate linear models for the analysis of RCT data is different from the usual interpretation of linear models. Table 2 explains the approach for interpreting the model which was re-coded and re-run to obtain estimates for the two groups involved (intervention and control). The impact of time on staff perceptions of barriers to change is described for both the intervention and control groups as changing the coding produces different results.

To aid interpretation of significant estimates, mean VOCALISE scores will be examined post hoc, using the post estimation command *lincom,* in Stata 14*.* This command computes point estimates, standard errors, *p*-values, and confidence intervals for the linear combination. These are based on the model, which adjusts for baseline differences, and therefore both groups have the same baseline score. A table which shows how staff perceived barriers to change at baseline according to the individual items of the VOCALISE measure is also presented (Appendix).

## Results

Overall, the analyses revealed that nursing staff who participated in the DOORWAYS intervention had significantly worse perceptions of barriers to change at the twelve month follow up, whilst the perceptions of those in the control group did not change over time. In both the intervention and control groups (*N* = 120), both ward and occupational status affected staff perceptions of barriers to change. However, across time, direct care staff in the intervention group exhibited more negative perceptions of barriers to change than those in the control group.

Given all staff with baseline data were included in the analysis whether or not they had follow up data, and those with follow up data were limited, the most representative picture of the sample characteristics can be seen at baseline. Table 3 describes the baseline characteristics of the staff participants from 8 wards. Wards differed in terms of the number of participants and the range of grades represented.

**Table 3:** Characteristics of the baseline participants [27]

Wards		1	2	3	4	5	6	7	8	Total (%)
N=	No. of staff	18 (15)	13 (10)	16 (12)	8 (6)	19 (15)	15 (12)	18 (15)	18 (15)	125 (100)
Staff Grade	HCA	7	3	6	1	5	4	7	6	39 (31)
	Band 5	8	7	7	3	12	8	7	6	58 (47)
	Band 6	1	2	1	3	1	0	3	4	15 (12)
	Band 7	1	1	1	1	1	1	1	1	8 (6)
	missing	1	0	1	0	0	2	0	1	5 (4)
Ethnic Group	White British /Other	6	2	3	4	5	3	4	6	33 (27)
	BME	12	11	12	4	14	12	14	10	89 (71)
	missing	0	0	1	0	0	0	0	2	3 (2)
Gender	Male	3	7	12	0	9	3	9	3	46 (37)
	Female	15	6	4	8	10	12	9	15	79 (63)
Age	Mean	39.63 (13.0)	36.38 (7.61)	38 (7.93)	44.25 (4.80)	43.26 (9.94)	35.38 (8.82)	39.6 (8.61)	40.07 (9.85)	39.57
	max/ min	27–50	22–62	24–55	37–49	26–67	22–48	27–55	23–54	N/A

Note: Band 7 staff are team leaders, band six staff are clinical charge nurses, band five staff are entry level qualified nursing staff and HCA’s are health care assistants

Table 4 outlines the numbers of staff who participated at follow up, providing repeated measures.

**Table 4:** The repeated measures sample numbers only

Repeated measures sample									
Ward	1	2	3	4	5	6	7	8	Total (%)
Number of staff	4	10	3	3	12	3	9	10	54 (100)
Group (INT/CTL)	CTL	CTL	INT	INT	INT	CTL	CTL	INT	INT:28 (52) CTL:26 (48)

Although all repeat participants remained on the same ward at both baseline and follow-up, shift rotations and sickness absences had the largest impact on follow-up participation. By using unstructured multivariate linear models the number of cases included in the final analyses (shown below in Table 5) was *N* = 120. At baseline, more than 60% of staff identified the following barriers (which are original items from the VOCALISE measure [27]) in 8 areas (detailed in Appendix):

- We can easily fit new changes in with our usual ward practices (72% agreed)
- I feel disheartened when others do not want to get involved in changes (77% agreed)
- I think that managing risk is more important than delivering new changes (64% agreed).
- I find it de-motivating when new changes do not take patients’ wishes into account (86% agreed).
- I think that some staff would rather let others take the lead in making changes (79% agreed).
- When some staff stop engaging with planned changes resistance spreads through my whole team (65% agreed).
- Inadequate staffing prevents changes being successful on my ward (89% agreed).
- Poor leadership prevents changes happening on my ward (61% agreed).

**Table 5:** Unstructured multivariate linear model (N = 120, 8 wards) exploring whether participation in the intervention affected staff perceptions of barriers to change, adjusting for time, ward and occupational status

Variables			Coef. β	S.E.	*P* Value	95%C.I.	
						LL	UL
*Intervention effect*			−5.16	2.62	0.05	−10.30	−0.02
*Time*			5.39	1.84	0.003	1.78	9.00
*Ward*	CTRL	Ward 2	−0.45	3.83	0.91	−7.95	7.05
	INT	Ward 3	−6.31	3.72	0.09	−13.60	0.98
	INT	Ward 4	−11.12	4.57	0.01	−20.08	−2.16
	INT	Ward 5	−12.06	3.50	0.001	−18.91	−5.21
	CTRL	Ward 6	−9.67	3.88	0.01	−17.27	−2.07
	CTRL	Ward 7	−8.14	3.53	0.02	−15.07	−1.22
	INT	Ward 8	−7.48	3.64	0.04	−14.61	−0.35
*Occupational status: manager/direct care staff*			−4.91	2.45	0.04	−9.71	−0.11
_cons			69.81	2.56	0	64.79	74.83

### Unstructured multivariate linear model exploring impact of the DOORWAYS intervention on staff perceptions of barriers to change, including covariates time, occupational status and ward

The impact of the intervention effect, time, occupational status and ward on VOCALISE were tested in an unstructured multivariate model (Table 5).

Overall this model was significant (χ^2^ (10) = 31.48; *p* > 0.001).

#### Intervention effect

In this model, the constant is the predicted mean score at baseline, if study group = 0 (control) and time is 0 and occupational status = 0 (or 1 for ward). The constant is the same for both the control and intervention groups, because a zero treatment effect is enforced at baseline. This meets the assumption of an RCT that there is no difference between scores at baseline, because any actual difference is assumed to exist by chance.

Perceptions of barriers to change were significantly higher (and therefore more negative) in the intervention group than the control group at follow up, after adjusting for all other covariates. At follow up, the estimate for the intervention effect variable shows that the predicted mean score in the intervention group was 5.16 more than the predicted mean score in the control group. This interpretation was not affected by reversing the coding.

#### Time

There was a difference in the way that the two groups responded to change over time. There was evidence (*p* = 0.003) of a change (adjusted for all other included covariates) in the estimated mean outcome score between baseline and follow up, in the intervention group. Over time, the scores in the intervention group became significantly worse because they increased by 5.39 points. The predicted mean outcome score in the intervention group at follow up was (75.20; C.I: 69.02 to 81.38). There was no significant change over time in the control group (Coef β: 0.23; S.E: 1.86; *p* = 0.90; C.I: − 3.42 to 3.89), if the model was rerun, changing the coding. The predicted mean outcome score in the control group at follow up was (70.04; C.I: 64.01to 76.07), showing little change from the baseline score.

#### Covariates

Occupational status significantly affected staff perceptions of barriers to change across time (*p* = 0.05), after adjusting for all other predictors. Post hoc, the mean predicted perceptions of barriers to change for those in direct care positions were more negative than those in more senior positions (Table 6).

**Table 6:** Predicted mean estimates for staff perceptions of barriers to change, according to (1) occupational status and (2) the interaction between time and occupational status

Mean predicted perceptions of barriers to change according to:	Study group (95% C.I.)	Time	Direct care staff	Senior staff
1. Occupational status only	Both groups	0	69.77 (64.75 to 74.79)	65.40 (58.80 to 71.99)
	Control group	FU	70.85 (64.66 to 77.03)	63.23 (55.08 to 71.37)
	Intervention group	FU	75.85 (69.56 to 82.13)	68.23 (59.76 to 76.69)
2. Occupational status*time	Both groups	0	69.77 (64.75 to 74.79)	65.40 (58.80 to 71.99)
	Control group	FU	70.85 (64.66 to 77.03)	63.23 (55.08 to 71.37)
	Intervention group	FU	75.85 (69.56 to 82.13)	68.23 (59.76 to 76.69)

Note: FU = follow up

The model does not explain whether the direct care staff perceptions of barriers to change grew more negative as a result of participation in the intervention. However, adding an interaction between time and occupational status showed a significant effect at follow up, in both groups (Coef β: -7.62; S.E: 3.55; *p* = 0.03; C.I:-14.59 to − 0.65). Table 6 shows more negative change in the perceptions of direct care staff in the intervention group.

The estimate for ward shows that there was a direct effect of certain wards on the outcome across time. The staff on ward 1, which was the reference category and a control ward, had the most negative perceptions as indicated by the constant (69.81). There was a significant difference between the reference ward and wards 4 to 8, which shows that perceptions of barriers to change varied by ward.

The adjusted mean outcome scores were computed post hoc (Table 7), which showed that perceptions on the intervention wards became more negative across time than those on the control wards.

**Table 7:** Mean estimates for staff perceptions of barriers to change by ward

Ward	Estimated Mean VOCALISE score at T0 (95%C.I.)	Estimated Mean VOCALISE score at follow up (95%C.I.)
1 (CTRL)	69.81 (64.79 to 74.83)	70.04 (64.01 to 76.07)
2 (CTRL)	69.36 (63.62 to 75.10)	69.59 (63.32 to 75 86)
6 (CTRL)	60.14 (54.39 to 65.89)	60.37 (53.61 to 67.13)
7 (CTRL)	61.67 (56.73 to 66.60)	61.90 (56.14 to 67.65)
3 (INT)	*63.50 (58.15 to 68.86)*	*68.89 (62.59 to 75.19)*
4 (INT)	*58.69 (51.08 to 66.30)*	*64.08 (55.89 to 72.26)*
5 (INT)	*57.75 (53.00 to 62.50)*	*63.14 (57.61 to 68.68*
8 (INT)	*62.33 (57.11 to 67.55)*	*67.72 (61.85 to 73.59)*

Including an interaction between ward and time was not possible in the model because there were a limited number of participants per ward.

## Discussion

Although the Nursing & Midwifery Council in the United Kingdom states that nursing staff should practice in line with the best available evidence [43], there is still a prominent disconnect between frontline practice and research evidence. Previous attempts to improve the uptake of research evidence into healthcare practice have generally targeted service users to adopt new interventions. This means that the role of ward staff in innovation has not been adequately investigated.

This study was part of an RCT (DOORWAYS) to improve the therapeutic milieu by delivering predominantly nurse led, CBT-based interventions. Although DOORWAYS had a positive impact on involuntary service user perceptions of, and satisfaction with, mental health wards [8, 9], there were also negative side effects from the changes, as staff perceptions of barriers to change worsened in those who participated in the intervention group. This finding provides support for the theories of Lewin [23] and Schein [24], who suggested that change brings disruption that can create resistance amongst staff. As well as barriers which were linked to resourcing issues, such as staffing, a number of the barriers perceived by staff at baseline also reflected a sense of demotivation amongst the workforce, an issue which may have perpetuated in the intervention group as the implementation process progressed (see Appendix, Table 8).

Given DOORWAYS was an externally devised change delivered in the form of a randomised controlled trial that was imposed at the ward level using a top down approach, it is perhaps unsurprising that staff responded negatively. This finding is in line with the wider management and heath literature which shows that changes implemented using a top down approach, with little input from front line staff can produce negative outcomes of increased stress, reduced job satisfaction, reduced psychological well-being and lower motivation [44, 45].

However, DOORWAYS did involve nursing stakeholders, and many of the practical suggestions made by frontline staff were adopted at the implementation stage. Senior ward staff (but not frontline staff, who were expected to implement the changes) were involved in discussion about the project upfront. Feedback from frontline staff was incorporated into the strategy via the psychologist who helped staff to set up each wards groups and also provided training, support and leadership to staff to enhance the learning process. It may be, therefore, that these findings reflect insufficient front line staff involvement or the high levels of nursing input required by the DOORWAYS intervention, in addition to their other tasks, with no time/resource allowance for that. As resources are limited in mental health wards, additional support may be required if complex RCTs are to be conducted in mental health wards in the future. Policy makers, National Health Service trusts and higher education settings might give further thought to how resources can be better allocated, given RCTs bring valuable learning experiences, which develop frontline staff, as well as measureable improvements and increased funding.

Although developing and implementing changes at the local level can produce more positive responses in frontline staff [46], these types of studies are rare. Future research programmes that seek to deliver substantial changes may need to develop implementations strategies which incorporate much greater stakeholder involvement, which could also be formally assessed over the period of change. This would also allow an exploration of whether more active stakeholder involvement might improve how staff regard barriers to change on the wards. In this way, feasibility issues might be addressed by encouraging staff generated adaptations that better suit the clinical environment.

Including contextual covariates in the model provided additional information to show that both occupational status and ward are involved in how staff respond to changes. In both groups, direct care staff had more negative perceptions of barriers to change than more senior staff. These findings are in line with previous literature [17, 25]. In both groups, the estimated mean VOCALISE scores on each ward at T1 revealed that some wards had significantly different scores from the reference ward (ward 1, a control ward). Irrespective of group allocation, there were also staff with more positive perceptions of barriers to change, both at the outset and at follow-up. It may be that staff who are more optimistic at the outset, simply remain more positive throughout, which suggests that some staff are better at coping with changes than others.

The model was not able to explain whether the relationship between ward climate and participation in DOORWAYS resulted in a negative effect on perceptions of barriers to change. However, by including an interaction between occupational status and time, it was possible to show that the changes introduced by DOORWAYS had a more detrimental on direct care staff than managers, over the 12 moth time period. This finding extends the current literature, and suggests that future change programmes should be sensitive to those working in demanding, direct care roles, at the inception of change, and through the implementation phase, as this sub-group may need extra support.

This study highlights negative side effects in terms of worsened perceptions of barriers to change as a result of a Trust-wide planned change, in a sample of mental health ward nurses. However, there were some limitations. First, as only one type of change was explored the results may not explain how ward staff might react to other types of innovation.

In addition, as this research was conducted in one trust, our understanding of the impact of DOORWAYS is restrictive. To ensure that future findings are widely generalizable, more than one organisation should be sampled. To better understand the impact of ward on perceptions of barriers to change, a larger number of wards should be included.

In implementation studies which are concerned with barriers in complex settings, the need to include contextual variables can mean that very large amounts of data are necessary to fully understand the picture. The large dropout rates in staff measures prevented an exploration of the bigger implementation picture (from initial disruption to embedding and sustaining changes), which might have been provided by the month long, stepped-wedge design of the DOORWAYS trial. This study was limited to using data from the 12 month time point, where 4 control wards could be compared to 4 wards that had received the intervention. This also meant that the amount of exposure to change was different (either 6 months or 12 months) which was a limitation. Nonetheless, unstructured multivariate linear models were used to overcome the reduced sample size. This method may therefore usefully inform future implementation studies.

## Conclusion

This study showed that participation in a clinical trial (DOORWAYS), which represented a period of intense change, had a negative impact on mental health ward staff perceptions of barriers to change. Given the large and often stressful workloads of mental health ward staff, careful thought should be given to supportive change management strategies that take the perspective of these staff into account. Involving staff in the development of research initiatives early on, may help to reduce resistance later on. Research programmes should also consider including a flexible implementation strategy, which may be informed by ward staff, to assess the impact and reduce the burden of changes.

In addition, occupational status (being from the direct care group) worsened staff perceptions of barriers to change in both the intervention and control groups. However, those in the intervention group became more negative in their views of change across time, having participated in DOORWAYS. This suggests that staff who provide direct care should be offered extra support when changes are introduced.

## Data Availability

The datasets used and/or analysed during the current study are available from the corresponding author on reasonable request.

## Appendix (Table 8)

**Table 8:** What were the barriers to change at baseline, according to staff perceptions?

Item number (N=)	Type of item	Total “disagree” responses (%)	Total “agree” responses (%)
1 (*N* = 124) When it comes to change, information is not circulated effectively on my ward.	BARRIER	59 (48)	65 (52)
*2 (N = 122) I feel confident when delivering new changes.*	*ENABLER*	*15 (12)*	*107 (88)*
*3 (N = 123) My whole team is regularly consulted about new ideas for ward practices.*	*ENABLER*	*20 (16)*	*103 (84)*
4 (N = 124) I’m too busy to keep up to date with information about the changes that are happening on my ward.	BARRIER	84 (68)	40 (32)
*5 (N = 123) We can easily fit new changes in with our usual ward practices.*	*ENABLER*	*88 (72)*	*35 (28)*
6 (N = 124) I feel disheartened when others do not want to get involved in changes.	BARRIER	28 (23)	96 (77)
7 (*N* = 121) I think that managing risk is more important than delivering new changes.	BARRIER	43 (36)	78 (64)
8 (N = 124) Changes just increase my workload and make my life harder.	BARRIER	81 (65)	43 (35)
9 (N = 122) It is not clear how all changes that we are asked to make will really benefit my ward.	BARRIER	56 (46)	66 (54)
10 (N = 124) My teammates think that there is no point trying to implement some changes because they won’t work.	BARRIER	75 (60)	49 (40)
11 (N = 123) I find it de-motivating when new changes do not take patients’ wishes into account.	BARRIER	17 (14)	106 (86)
12 (N = 121) I think that some staff would rather let others take the lead in making changes.	BARRIER	26 (21)	95 (79)
13 (N = 120) When some staff stop engaging with planned changes resistance spreads through my whole team.	BARRIER	42 (35)	78 (65)
14 (N = 123) I do not really understand how to deliver some of the changes that are suggested by the management.	BARRIER	76 (62)	47 (38)
*15 (N = 119) Changes are audited to increase their consistent delivery on my ward.*	*ENABLER*	*19 (16)*	*100 (84)*
*16 (N = 121) I always challenge team members who are avoiding delivering new changes.*	*ENABLER*	*43 (36)*	*78 (64)*
17 (N = 123) Inadequate staffing prevents changes being successful on my ward.	BARRIER	13 (11)	110 (89)
18 (N = 123) Poor leadership prevents changes happening on my ward.	BARRIER	48 (39)	75 (61)

Note: VOCALISE items were answered using a Likert scale (Strongly Agree = 1, Agree = 2, Slightly Agree = 3, Slightly Disagree = 4, Disagree = 5, Strongly Disagree = 6), however for simplicity answers are presented here in terms of positive/negative responses

## Abbreviations

CBT: Cognitive Behavioural Therapy
NHS: National Health Service
RCT: Randomised Controlled Trial
VOCALISE: View Of Change and Limitations in In-patient Settings

## References

[1] Eccles MP, Armstrong D, Baker R, Cleary K, Davies H, Davies S (2009). An implementation research agenda. Implement SciImplement Sci.

[2] Grol RP, Bosch MC, Hulscher ME, Eccles MP, Wensing M (2007). Planning and studying improvement in patient care: the use of theoretical perspectives. Milbank Q.

[3] Williams N, Glisson C, Hemmelgarn A, Green P (2017). Mechanisms of Change in the ARC organizational strategy: increasing mental health clinicians EBP adoption through improved organizational culture and capacity. Adm Policy Ment Health Ment Health Serv Res.

[4] Corry P (2004). Behind closed doors – acute mental health care in the UK.

[5] Evans J, Rose D, Flach C, Csipke E, Glossop H, McCrone P (2012). VOICE: developing a new measure of service users’ perceptions of inpatient care, using a participatory methodology. J Ment Health.

[6] Csipke E, Flach C, McCrone P, Rose D, Tilley J, Wykes T (2013). Inpatient care 50 years after the process of deinstitutionalisation. Soc Psychiatry Psychiatr Epidemiol.

[7] Sharac J, McCrone P, Sabes-Figuera R, Csipke E, Wood A, Wykes T (2010). Nurse and patient activities and interaction on psychiatric inpatients wards: a literature review. Int J Nurs Stud.

[8] Wykes T, Csipke E, Williams P, Koeser L, Nash S, Rose D (2018). Improving patient experiences of mental health inpatient care: a randonmised controlled trial. Psychol Med.

[9] Wykes T, Csipke E, Rose D (2018). Patient involvement in improving the evidence base on mental health inpatient care: the PERCEIVE programme.

[10] Rose D, Evans E, Laker C, Wykes T (2015). Life in acute mental health settings: experiences and perceptions of service users and nurses. Epidemiol Psychiatr Sci.

[11] Powell BJ, Waltz TJ, Chinman MJ, Damschroder LJ, Smith JL, Matthieu MM (2015). A refined compilation of implementation strategies: results from the Expert Recommendations for Implementing Change (ERIC) project. Implement Sci.

[12] Alexander JA, Hearld LR (2012). Methods and metrics challenges of delivery-system research. Implement Sci.

[13] Priebe S, Badesconyi A, Fioritti A, Hansson L, Kilian R, Torres-Gonzales F (2005). Reinstitutionalisation in mental health care: comparison of data on service provision from six European countries. BMJ.

[14] Landaeta R, Mun JH, Rabadi G (2008). Identifying sources of resistance to change in healthcare. Int J Healthc Technol Manag.

[15] Schoenwald SK, Carter RE, Chapman JE, Sheidow AJ (2008). Therapist adherence and organizational effects on change in youth behavior problems one year after multisystemic therapy. Admin Pol Ment Health.

[16] Cleary M (2004). The realities of mental health nursing in acute inpatient environments. Int J Ment Health Nurs.

[17] Brennan G, Flood C, Bowers L (2006). Constraints and blocks to change and improvement on acute psychiatric wards--lessons from the city nurses project. J Psychiatr Ment Health Nurs.

[18] Totman J, Hundt GL, Wearn E, Paul M, Johnson S (2011). Factors affecting staff morale on inpatient mental health wards in England: a qualitative investigation. BMC Psychiatry.

[19] Bowers L (2014). Safewards: a new model of conflict and containment on psychiatric wards. J Psychiatr Ment Health Nurs.

[20] Kindy D, Petersen S, Parkhurst D (2005). Perilous work: nurses’ experiences in psychiatric units with high risks of assault. Arch Psychiatr Nurs.

[21] Kuokkanen L, Suominen T, Harkonen E, Kukkurainen ML, Doran D (2009). Effects of organizational change on work-related empowerment, employee satisfaction, and motivation. Nurs Adm Q.

[22] Martin A, Jones ES, Callan VJ (2005). The role of psychological climate in facilitating employee adjustment during organisational change. Eur J Work Organ Psychol.

[23] Lewin K (1951). Field theory in social science.

[24] Schein E (1996). Kurt Lewin’s change theory in the field and in the classroom: notes toward a model of managed learning. Syst Pract.

[25] Benn J, Burnett S, Parand A, Pinto A, Iskander S, Vincent C (2009). Perceptions of the impact of a large-scale collaborative improvement programme: experience in the UK safer patients initiative. J Eval Clin PractJ Eval Clin Pract.

[26] Laker C, Callard F, Flach C, Williams P, Sayer J, Wykes T (2014). The challenge of change in acute mental health services: measuring staff perceptions of barriers to change and their relationship to job status and satisfaction using a new measure (VOCALISE). Implement SciI.

[27] Laker C, Cella M, Callard F, Wykes T (2019). Why is change a challenge in acute mental health wards? A cross-sectional investigation of the relationships between burnout, occupational status and nurses’ perceptions of barriers to change. Int J Ment Health Nurs.

[28] Laker C, Cella M, Callard F, Wykes T (2019). The impact of ward climate on staff perceptions of barriers to research driven service changes on mental health wards: a cross sectional study. J Psychiatr Ment Health Nurs.

[29] Penn D, Roberts DL, Combs D, Serne A (2007). The development of the social cognition and interaction training program for schizophrenia spectrum disorders. Psychiatr Serv.

[30] Ruddle A, Mason O, Wykes T (2011). A review of hearing voices groups: evidence and mechanisms of change. Clin Psychol Rev.

[31] Knight MTD, Wykes T, Hayward P (2006). Group treatment of perceived stigma and self esteem in schizophrenia: a waiting list trial of efficacy. Behav Cogn Psychother.

[32] Lineham M (1997). Skills training manual for the treatment of borderline personality disorder.

[33] Grey S (2015). Problem solving groups for psychiatric inpatients: a practical guide.

[34] Rogers EM (2003). Diffusion of innovations.

[35] Green SB (1991). How many subjects does it take to do a regression analysis?. Multivar Behav Res.

[36] Frost C, Kenward M, Fox N (2008). Optimizing the design of clinical trials where the outcome is a rate. Can estimating a baseline rate in a run-in period increase efficiency?. Stat Med.

[37] Laird N, Waire J (1982). Random-effects models for longitudinal data. Biometrics.

[38] Molenberghs G, Kenward MG (2007). Missing data in clinical studies.

[39] White I, Thompson S (2005). Adjusting for partially missing baseline measurements in randomized trials. Stat Med.

[40] Carpenter JR, Kenward MG (2008). Missing data in randomised controlled trials – a practical guide.

[41] Rubin DB (1976). Inference and missing data. Biometrika.

[42] Carpenter J, Kenward M (2013). Multiple imputation and its application.

[43] NMC (2018). The Code: Professional standards of practice and behaviour for nurses, midwives and nursing associates.

[44] Diefenbach T (2009). New public management in public sector organisations: the dark sides of managerialistic ‘enlightenment’. Public Adm.

[45] Teo S, Yeung M, Change E (2011). Administrative stressors and nursing job outcomes in Australian public and non-profit health care organisations. J Clin Nurs.

[46] Taylor-Watt J, Cruikshank A, Innes J, Brome B, Shah A (2017). Reducing physical violence and developing a safety culture across wards in East London. Br J Ment Health Nurs.

